# Protrusion pyramidale périanale

**DOI:** 10.11604/pamj.2014.18.224.4758

**Published:** 2014-07-17

**Authors:** Nihal Bekkali, Jalal El Benaye

**Affiliations:** 1 Service de Dermatologie, CHU Avicenne Rabat, Maroc; 2 Service de Dermatologie Hôpital militaire, Maroc

**Keywords:** Protrusion pyramidale, anus, constipation, pyramidal protrusion, anus, constipation

## Image en medicine

**Figure 1:**
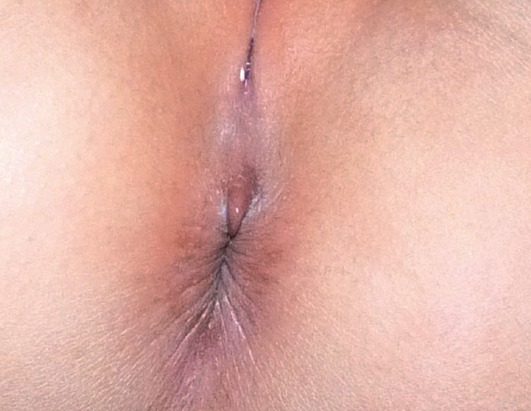
Lésion saillante pyramidale rosée Au niveau de la partie antérieure du sillon ano-périnéal

La protrusion pyramidale périanale est une entité d'individualisation récente, assez rare, touchant presque exclusivement le sexe féminin souvent en bas âge, d'origine congénitale, fonctionnelle ou associée au lichen scléroatrophique. Dans la première forme, la lésion serait due à une faiblesse constitutionnelle de la paroi périnéale, notamment du raphé médian, ou serait secondaire à une malformation du septum urogénital lors de la vie embryonnaire. Ceci expliquerait l'atteinte exclusive du sexe féminin et la présence de cas familiaux. La forme fonctionnelle serait secondaire surtout à la constipation mais aussi à la diarrhée et aux fissures anales. Certains auteurs suggèrent le rôle déclenchant ou entretenant des irritations locales. Les lésions cutanées sont le plus souvent prises à tord pour des condylomes, des vestiges anaux ou encore des sévices sexuels. L'évolution est bénigne avec souvent une régression spontanée sur plusieurs semaines. Nous rapportons l'observation d'un nourrisson de sexe féminin, âgé d'un an, asymptomatique. L'examen clinique objectivait au niveau de la partie antérieure du sillon ano-périnéal une lésion saillante cutanéo-muqueuse de 5 mm de diamétre, grossièrement pyramidale, de couleur rosée et à surface lisse légèrement veloutée (Figure 1). Il n'y avait ni irritation, ni fissuration, ni troubles pigmentaires, ni cas similaire dans la famille. L'interrogatoire rapportait une notion de trouble du transit à type de constipation, depuis 2 semaines. Le diagnostic de protrusion pyramidale péri anale liée à la constipation ainsi posé, un régime riche en fibres est instauré avec reprise d'un transit normal sans amélioration de la lésion cutanée après un recul de six semaines.

